# Thai version of the dry eye-related quality-of-life score questionnaire: preliminary assessment for psychometric properties

**DOI:** 10.1186/s12886-021-02077-0

**Published:** 2021-08-28

**Authors:** Sasiwimon Tansanguan, Napaporn Tananuvat, Nahathai Wongpakaran, Tinakon Wongpakaran, Somsanguan Ausayakhun

**Affiliations:** 1 Department of Ophthalmology, Faculty of Medicine, Chiang Mai University, Chiang Mai, 50200 Thailand; 2 Department of Psychiatry, Faculty of Medicine, Chiang Mai University, Chiang Mai, 50200 Thailand

**Keywords:** Dry eye, Quality-of-life, Questionnaire, Reliability, Thai, Validity

## Abstract

**Background:**

This study aimed to develop a Thai version of the Dry Eye–Related Quality-of-Life Score (DEQS-Th) questionnaire and evaluate its validity, reliability, and feasibility among Thai participants.

**Methods:**

The DEQS-Th, a 15-item self-report measuring dry eye and its impact on quality of life (QOL) was developed based on the DEQS. The questionnaire was divided into two subscales: *Bothersome Ocular Symptoms* (six questions), and *Impact on Daily Life* (nine questions). It employed a 5-point Likert scale, addressing on both the frequency and the degree of symptoms. Backward and forward and cultural adaptation process translation methods were employed. Thirty healthy participants were enrolled to evaluate the feasibility of the DEQS-Th in terms of difficulty and convenience. Reliability was assessed using internal consistency determined by Cronbach’s alpha, with values > 0.7 considered acceptable. Convergent validity was determined by the correlation between DEQS-Th and overall health status. Confirmatory factor analysis was conducted for its factor structure.

**Results:**

The participants’ mean age was 38.6 ± 12.9 years, and 23 (76.7%) were females. The mean time to complete the questionnaire was 9.3 ± 2.7 min. The Cronbach’s alpha of the ocular symptoms subscale, impact on QOL subscale, and summary score on frequency and degree were 0.80 and 0.70, 0.89 and 0.89, and 0.90 and 0.89, respectively. The overall health status significantly correlated with the summary score (*r* = 0.564, *p* = .001), subscale ocular symptoms (*r* = 0.594, *p* = .001), and impact on QOL scores (*r* = 0.626, *p* < .001) of the DEQS-Th, respectively. A one-factor model fitted the data the best for both the ocular symptoms subscale (CFI = 1.000, TLI = 1.000, RMSEA = 0.000) and the impact on QOL subscale (CFI = 0.998, TLI = 0.997, RMSEA = 0.053).

**Conclusion:**

When tested among normal participants, the DEQS-Th is a valid and reliable measurement for dry eye symptoms and impact on QOL.

**Supplementary Information:**

The online version contains supplementary material available at 10.1186/s12886-021-02077-0.

## Background

Dry eye disease (DED) is a multifactorial disease of the ocular surface, including loss of homeostasis of the tear film, ocular symptoms, tear film instability and hyperosmolarity, ocular surface inflammation and damage, and neurosensory abnormalities [1]. DED is recognized as a common and growing eye problem worldwide. Its prevalence, with and without symptoms, ranges from 5 to 50% [1]. The prevalence, based on signs alone, is generally higher and varied, reaching up to 75% in some studies [1, 2]. This may reflect the diversity between symptoms and clinical findings. However, dry eye symptoms such as pain, discomfort, and visual disturbance are common, resulting in complaints from patients seeking eye care. These symptoms have a major impact on treatment outcomes [3, 4].

Chronic symptoms of DED can affect the quality of life (QOL) of patients. Evidence that DED can diminish QOL in the affected population is steadily increasing [5]. Related studies have shown that moderate-to-severe dry eye can cause a decrease in patients’ QOL comparable to that in renal dialysis and severe angina [6, 7]. Accordingly, measurement of the impact of DED on patients’ daily lives is now recognized as a critical aspect of disease characterization.

In its 2017 epidemiologic report on DED, the International Dry Eye Workshop highlighted several questionnaires; however, few of these questionnaires provided information regarding both symptoms and health-related QOL among patients with dry eye. The Ocular Surface Disease Index (OSDI) and the Impact of Dry Eye on Everyday Living (IDEEL) are dry eye–specific questionnaires that have been validated and used frequently [1]. The OSDI is a 12-item questionnaire that assesses both dry eye symptoms and their effects on vision-related functioning over a week. It contains three subsections including vision-related function, ocular symptoms, and environmental triggers. The IDEEL is a three-module questionnaire with 57 questions that assesses dry eye symptoms, the effects of dry eye on QOL, and treatment satisfaction after 2 weeks [8]. One of the limitations of the OSDI is that it addresses only some of the effects of DED on visual-related function, and it does not address the effects of DED on patients’ daily lives. However, the OSDI can be completed much more quickly and is free [9]. The primary limitation of the IDEEL is that it is time-consuming and must be purchased, which may be a restriction in clinical practice [9]. The other two questionnaires that are widely used regarding general health and eye health include the Short Form 36 and the 25-item National Eye Institute’s Visual Function Questionnaire (NEI VFQ-25), respectively [1]. However, neither are disease-specific, requiring further validation and reliability testing in a dry eye cohort.

In 2013, the Dry Eye–Related Quality-of-Life Score (DEQS) questionnaire was developed and validated in Japan [10]. This questionnaire consisted of 15 items and two subscales: *Bothersome Ocular Symptoms* and *Impact on Daily Life*. Compared to results from the Short Form-8 Health Survey and the NEI VFQ-25, the results from the DEQS were valid and reliable for assessing the effects of DED on QOL, including mental health. This suggests that this measurement may be used in routine clinical practice.

Other newly developed health-related QOL questionnaires for patients with DED include the University of North Carolina Dry Eye Management Scale (UNC DEMS) and the Chinese version of the Dry Eye–Related Quality of Life (CDERQOL) [11]. The UNC DEMS is a single-item questionnaire developed in 2014 that has good reliability and validity and is strongly correlated with the OSDI [11, 12]. The CDERQOL was created in PR China based on the IDEEL questionnaire developed in 2017. This 45-item questionnaire has good psychometric properties, including construct validity and internal consistency (Cronbach’s alpha = 0.72–0.91) [13].

Similar to the reported prevalence of DED in other Asian countries [2], DED also involves common eye problems up to 34%, as reported in the prevalence of DED in Thailand [14]. Nevertheless, no study has been published regarding the effects of DED on QOL among Thai patients. This may be partly due to the limited tools regarding the dry eye-specific questionnaire in Thai that could assess the patients’ QOL. Therefore, this study aimed to develop a translated and cross-cultural adapted Thai version of the DEQS (DEQS-Th) and to evaluate its validity, reliability, and feasibility among healthy participants.

## Methods

This prospective cross-sectional study was approved by the Research and Ethics Committee, Faculty of Medicine, Chiang Mai University (Study code 010–2562), and followed the Declaration of Helsinki. All volunteers signed written informed consent forms, obtained after a complete explanation was provided.

### Dry eye–related quality-of-life score (DEQS)

The DEQS questionnaire consists of 15 questions divided in two subscales: the *Bothersome Ocular Symptoms* (six questions) and the *Impact on Daily Life* (nine questions) [10]. Each question is assessed using two-step scales. The first step is to assess the frequency of symptoms and disability, and the second is to assess the degree of severity. The frequency is scored on a 5-point Likert scale ranging from 0 to 4 (0 = *never*, 4 = *always*). When the answer was “never,” then the respondent could skip to the next question; but when any frequency was reported (1–4), the respondent had to rate the degree of severity, which was scored on a 4-point Likert scale ranging from 1 to 4, with a larger number indicating a greater burden. As recommended by the developer of the DEQS, the total score of the answer was calculated using the summation of the degree scores of all questions answered multiplied by 25 and divided by the total number of questions answered. The total score ranging from 0 to 100, with a higher score, represented greater disability. Subscale scores were calculated similarly, using only the item from each subscale.

The DEQS also provided the health status numerical questionnaire. The respondents were asked to rate their overall health status, from 0 (*extremely well*) to 6 (*extremely bad*), over the prior week, including the eye symptoms and how they had affected the respondents’ daily lives.

### Translation and cross-cultural adaptation process

**Fig. 1:**
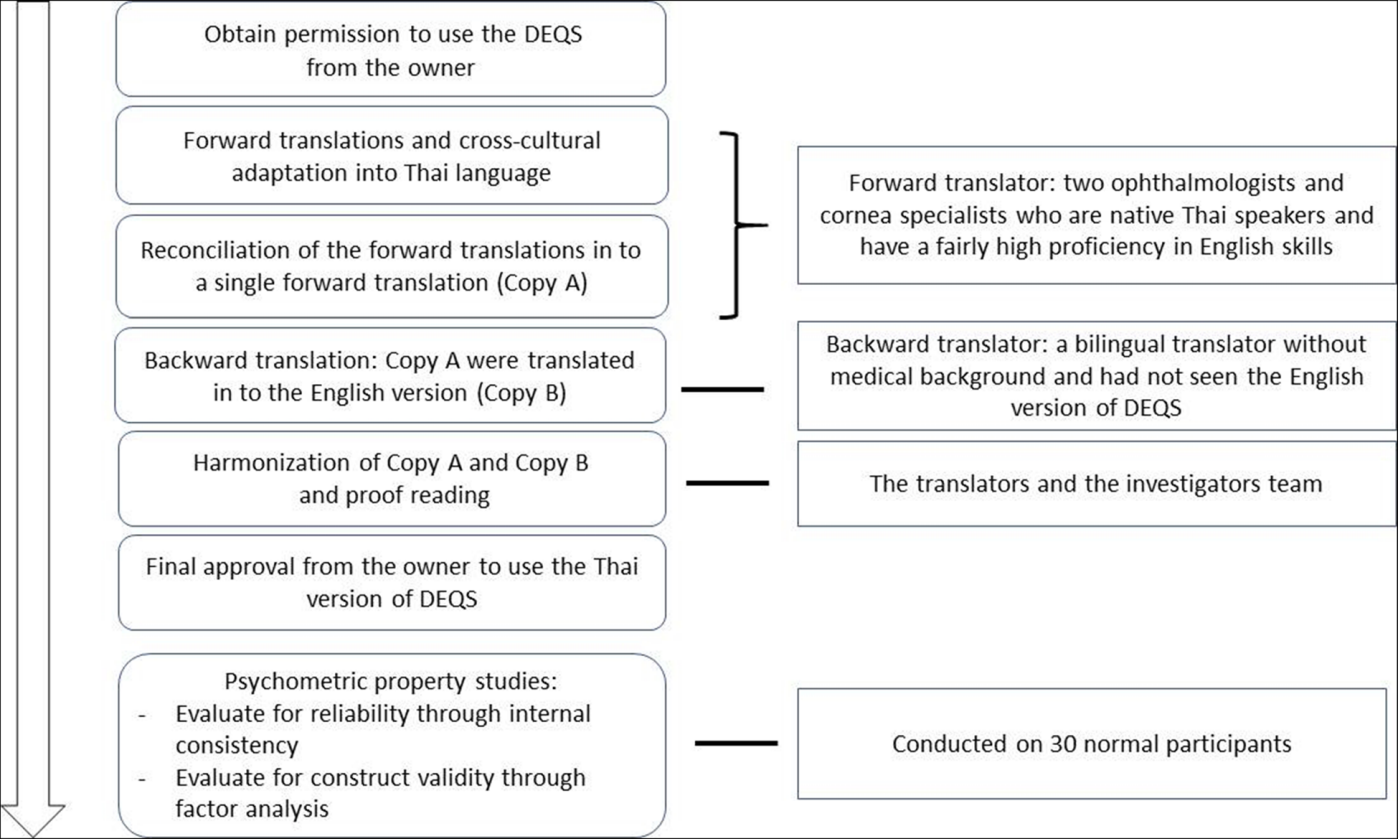
Development Process of the Thai Version of the Dry Eye-Related Quality-of-Life Score Questionnaire

After permission from the owner of the DEQS (the Asia Dry Eye Society and Santen Pharmaceutical Co., Japan) was granted, the translation and cross-cultural adaptation of the DEQS questionnaire to Thai was conducted according to principles of good practice reported by the International Society for Pharmaco-economics and Outcomes Research for translation and cultural adaptation [15] (Fig. 1).

#### Step 1: forward translations and cultural adaptation

The DEQS questionnaire was translated into Thai with cultural adaptation by two qualified Thai ophthalmologists and corneal specialists (NT, SA) who were proficient in written and spoken English. The translation was conducted individually, resulting in two forward translations. Then two forward translators were discussed and any discrepancies in the translations were resolved, resulting in one common forward translation (Copy A).

#### Step 2: backward translations

A bilingual translator without a medical background and who had not seen the English version of the DEQS translated the forward translated Thai version (Copy A) to a backward English translation (Copy B).

#### Step 3: consensus by the translators and the approval for use

To ensure conceptual equivalence between the original and the target language version, the authors and translators met and discussed discrepancies between the different versions until a consensus was reached. The Thai version (Copy A) and the backward translation version (Copy B) were sent to the DEQS’s owner for approval of the final version of the DEQS-Th.

### Internal consistency and feasibility of the DEQS-Th

#### Participants

The eligibility criteria included healthy individuals aged 18 or older with a Thai native speaker literate in Thai. Participants were excluded if they had best corrected visual acuity of less than 6/6, abnormalities in tear function, tear film break up time of fewer than 5 seconds, and general health problems or disabilities that affected their daily life, including any psychological disorders. Contact lens wearers were also excluded. All participants completed the DEQS-Th independently, and the time to complete the questionnaire for each participant was recorded in minutes.

#### Statistical analysis

The participants’ demographic data were descriptively analyzed. For numerical data, mean (SD) was used for normally distributed data, while the median (range) was used for non-normally distributed data. Internal consistency was calculated to evaluate the reliability of the questionnaire (frequency and degree). A Cronbach’s alpha coefficient of 0.7 or higher was considered acceptable. To test the validity, two methods were used: first, for convergent validity, Pearson’s correlation coefficient was used to evaluate the correlations between the overall health status and the summary and subscale scores of DEQS-Th; and second, construct validity (Fig. 1). Based on the related research [10], both symptom and impact on QOL subscales were considered as unidimensional constructs; therefore, confirmatory factor analysis was used to ensure the one-factor (unidimensional) model of the symptom subscale and the impact on the QOL subscale. To meet the criteria for unidimensionality, each item should sufficiently contribute to the same construct, indicated by the standardized loading factor more than 0.4 [16]. Ideally, the correlation between each item should not be too high, and the error term of each pair of items is expected not to be correlated. Weighted Least Square Mean and Variance (WLSMV) corrected method of estimation was used for the non-normality and ordinal types of items. Chi-square was used to evaluate a model fit, where *p*-values >.05 or chi-square/df < 3 were considered acceptable fits. Other fit indices included Comparative Fit Index (CFI) and Tucker-Lewis Index (TLI), where values of 0.95 or higher were preferable [17]. Root mean square error of approximation (RMSEA) was also performed, and a value < 0.08 was indicative of an acceptable model fit [18]. Despite the study employing a small sample size, the size was sufficient for factor analysis. Based on Mundfrom et al., the high communality level (0.6–0.8) with the ratio of variables to factors (six for the symptom subscale and nine for the impact on QOL subscale) required a sample size of at least 17–19 [19].

SPSS (Version 22.0, SPSS Inc., Chicago, IL, USA) was used to analyze the data. Mplus, Version 8.5, was used for confirmatory factor analysis (CFA).

## Results

Thirty healthy participants with a mean age of 38.6 ± 12.9 years, were enrolled. Among these, 23 (76.7%) were females. All worked indoors. The baseline characteristics of the participants are shown in Table 1. The mean time to complete the questionnaire was 9.3 ± 2.7 (range 3–15) minutes. Table 2 demonstrates the overall Cronbach’s alpha (frequency and degree) and that of the *Bothersome Ocular Symptoms* and *Impact on Daily Life* subscales. The mean score for the subscale ocular symptoms, subscale impact on daily life, and the summary scores of the DEQS-Th was 9.3 ± 7.9, 15.4 ± 15.7, and 14.8 ± 12.7, respectively. The median and range of the frequency and degree scores of each item and the Cronbach’s alpha of the subscale when each question was deleted are shown in Table 3. The overall health status significantly correlated with the summary score (*r* = 0.564, *p* = .001), *Bothersome Ocular Symptoms* scores (*r* = 0.594, *p* = .001), and *Impact on Daily Life* scores (*r* = 0.626, *p* < .001) of the DEQS-Th.

**Table 1:** Participant characteristics and self-rating overall health status

Characteristic	
Age (years)	
- Mean ± SD	38.6 ± 12.9
- Range	22–60
Sex (*n*, %)	
- Male	7 (23.3)
- Female	23 (76.7)
Career (*n*, %)	
- Indoors	30 (100.0)
Exercise (*n*, %)	
- Regularly	5 (16.7)
- Sometimes	19 (63.3)
- Rarely/Never	6 (20.0)
Systemic health problems (*n*, %)	
- None	20 (66.7)
- Diabetes mellitus	10 (33.3)
- Hypertension	2 (6.7)
- Dyslipidemia	3 (10.0)
- Systemic lupus erythematosus	3 (10.0)
- Osteoporosis	1 (3.3)
- Menopause	2 (6.7)
Smoking (*n*, %)	2 (6.7)
Overall health status^a^	
- Mean ± SD	2.53 ± 0.13
- Range	1–3

^a^ Range from 1 (extremely good) to 6 (extremely bad)

**Table 2:** Frequency and degree scores and Cronbach’s alpha in each subscale, and summary scores of the DEQS-Th

Subscale and summary score	Median score (range)		Cronbach’s alpha	
	Frequency	Degree	Frequency	Degree
*Bothersome Ocular Symptoms* subscale	2.0 (0–6)	4.0 (0–19)	0.80	0.70
*Impact on Daily Life* subscale	3.0 (0–9)	2.0 (0–9)	0.89	0.89
Summary score	5.0 (0–13)	7.0 (0–28)	0.90	0.89

**Table 3:** Median scores of each item and the Cronbach’s alpha of the subscale of the DEQS-Th when each item was deleted

All items and subscales	Median score (range)		Cronbach’s alpha	
	Frequency^**a**^	Degree^**b**^	Frequency	Degree
***Bothersome Ocular Symptoms***				
1. Foreign body sensation	1.0 (0–3)	1.0 (0–4)	0.75	0.67
2. Dry sensation in eyes	0.5 (0–3)	0.5 (0–2)	0.70	0.58
3. Painful or sore eyes	0.0 (0–2)	0.0 (0–2)	0.79	0.65
4. Ocular fatigue	0.5 (0–3)	0.5 (0–2)	0.74	0.61
5. Heavy sensation in eyelids	0.0 (0–3)	0.0 (0–2)	0.80	0.74
6. Redness in eyes	0.0 (0–1)	0.0 (0–2)	0.79	0.66
***Impact on Daily Life***				
7. Difficulty opening eyes	0.0 (0–2)	0.0 (0–2)	0.89	0.89
8. Blurred vision when watching something	1.0 (0–4)	1.5 (0–4)	0.86	0.87
9. Sensitivity to bright light	0.5 (0–3)	0.5 (0–3)	0.88	0.88
10. Problems with eyes when reading	1.0 (0–4)	1.5 (0–4)	0.87	0.87
11. Problems with eyes when watching television, looking at a computer or using a cell phone	1.0 (0–3)	1.0 (0–3)	0.86	0.87
12. Feeling distracted because of eye symptoms	0.0 (0–2)	0.0 (0–2)	0.88	0.88
13. Eye symptoms affecting work	0.0 (0–3)	0.0 (0–3)	0.87	0.88
14. Not feeling like going out because of eye symptoms	0.0 (0–2)	0.0 (0–2)	0.89	0.90
15. Feeling depressed because of eye symptoms	0.0 (0–2)	0.0 (0–2)	0.89	0.90

^a^ Range from 0 (*not at all*) to 4 (*always*), and ^b^ Range from 0 (*not at all*) to 4 (*very much*)

**Fig. 2:**
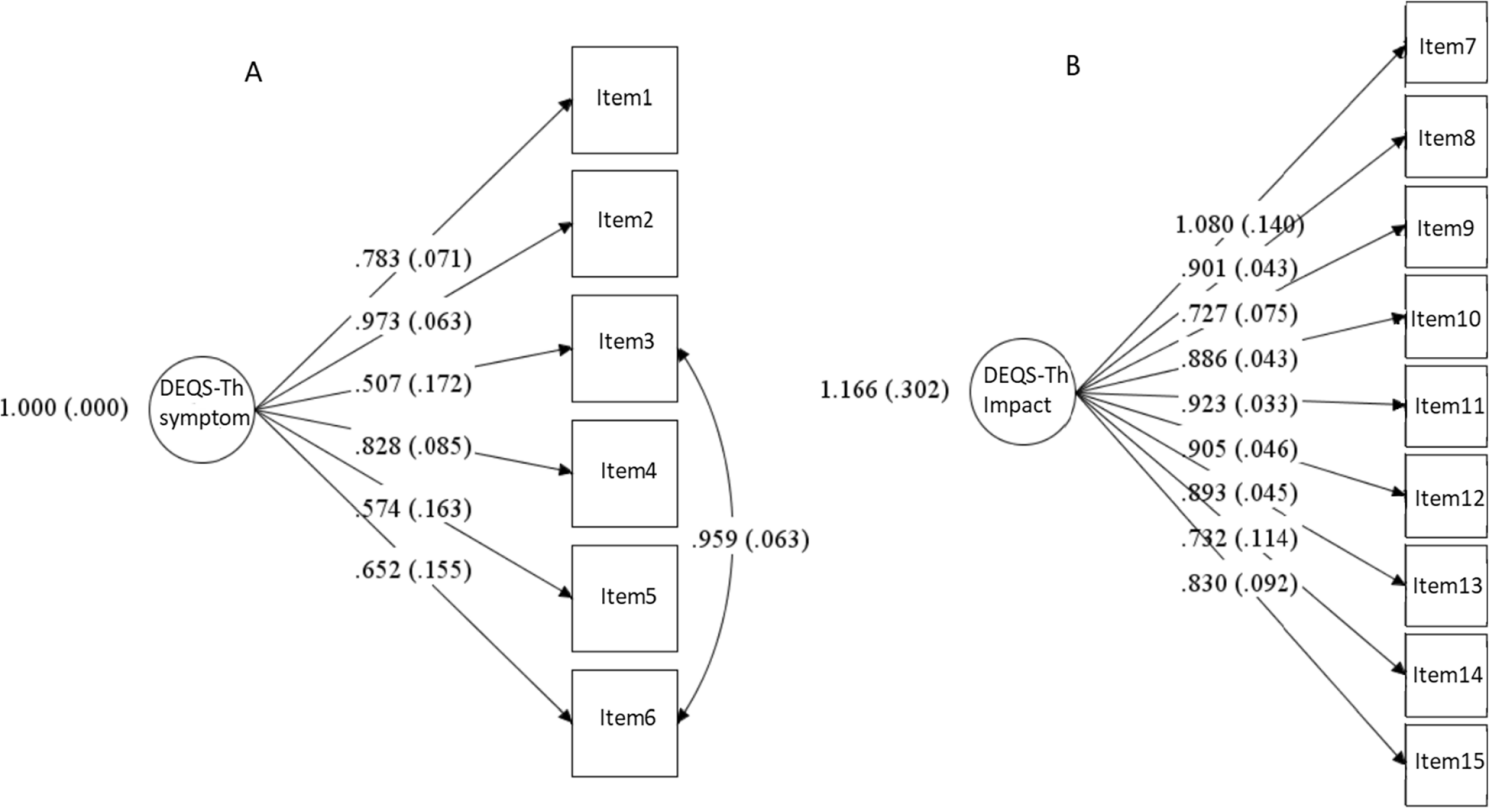
Results of the Confirmatory Factor Analysis for DEQS-Th Questionnaire. **A** the standardized factor loadings of each item on the symptom subscale, with covariance of error terms of item 3 and item 6; **B** the standardized factor loadings of each item on the impact on daily life subscale

Figure 2 shows the results of the CFA of each subscale. All items significantly loaded on the same construct, ranging from 0.51 to 0.83 (all *p* < .0001, except Item 3) for the symptom subscale, and from 0.73 to 1.08 (all *p* < .0001) for the impact on QOL subscale. For model fitness, the CFA of the symptom subscale showed a Chi-square of 4.439, df = 8, *p* = 0.816, CFI = 1.000, TLI = 1.000, RMSEA = 0.000 (90% CI [0.000–0.133]), indicating a unidimensional scale. However, the error terms of Items 3 and 6 were suggested to be correlated. CFA of the impact on daily life subscale showed a Chi-square of 29.317, df = 27, *p* = 0.346, CFI = 0.998, TLI = 0.997, RMSEA = 0.053 (90% CI [0.000–0.156]), indicating a unidimensional scale. (Supplement Table S1).

## Discussion

This study demonstrated the preliminary results of the Thai version of the Dry Eye–Related Quality-of-Life Score (DEQS-Th) questionnaire confirming it as a valid, reliable, and feasible tool. Compared with the DEQS in Japanese, the original version, and other versions in different languages, including English, French, Deutsch, Chinese, and Korean, the Thai version had good internal consistency when tested among the normal study sample. The Cronbach’s alpha values of the DEQS-Th were acceptable on the score of the *Bothersome Ocular Symptoms* subscale*,* and highly acceptable on the *Impact on Daily Life* subscale and the summary score. However, these values were slightly lower than those of the original version (0.83, 0.93, and 0.93, respectively) [10].

From a sensitivity analysis determining the Cronbach’s alpha of the subscale when each item was deleted, a low Cronbach’s alpha value was generally revealed pertaining to the symptoms subscale. This indicated that each item importantly contributed to the scale, except for three items: painful (Item 3), heavy sensation in eyelids (Item 5), and redness (Item 6). This was also in line with the CFA results showing the relatively low loadings of Items 3, 5, and 6. The CFA also suggested that Items 3 and 6 were highly related. This may have been due to the nonspecific cause of these ocular symptoms. This pair of items should be further investigated in a larger sample size. Another reason for a low Cronbach’s alpha was that the DEQS-Th in the present study was tested among healthy participants. Notably, the Cronbach’s alpha values of the *Bothersome Ocular Symptom* subscale were slightly lower than those of the *Impact on Daily Life* subscale. These findings were also supported by a related study concerning the reliability and validity of ODSI [20]. The ocular symptoms of patients with dry eye typically vary due to the fluctuation in subjective symptoms, which may reduce reliability.

Previously, the OSDI questionnaire—the only dry eye questionnaire available in Thai—has been used worldwide since 2000 [21]. The ODSI could evaluate both subjective DED symptoms and vision-related functioning, and has proven to be a valuable patient report outcome measure in clinical trials and practice [21]. Further, the OSDI can discriminating between normal, mild-to-moderate, and severe DED [21]. However, some limitations of the OSDI include a lack of common symptoms related to DED, such as “foreign body sensation” and “eye fatigue.” Some items in the OSDI are similar—such as “blurred vision” and “poor vision”—and could be related to other eye problems. In addition, some items on the environmental subscale of the OSDI, such as “windy conditions” and “place with low humidity” may not be applicable in some regions, especially in a tropical area like Thailand, where the weather is likely to be hot and humid year-round. Therefore, compared to the DEQS, the OSDI captures only some visual-related functioning but not the effects on patients’ daily lives. Moreover, OSDI responses are limited to only the frequency, not the severity, of the symptoms whereas the DEQS involves two-step scales (frequency and degree). Although endorsing the frequency of symptoms appears to be easier than that concerning the degree of severity, the degree scale may represent the patient’s burden and may be more appropriate for evaluating the effects of DED on daily life. Thus, the degree score is used for calculation in the DEQS questionnaire [10]. However, the DEQS may be cumbersome for respondants because it requires responding to up to 30 questions, compared with the 12 questions in the OSDI. Nevertheless, a recent study showed that the OSDI and the DEQS are significantly correlated, with negligible score differences [22]. This evidence affirms the use of the DEQS-Th as a valid method to assess subjective dry eye symptoms. Regarding the impact on QOL, all items were relevant and useful for this subscale, according to the CFA results.

The present study also showed that the DEQS-Th is a user-friendly measurement, as the time to complete the questionnaire was short and comparable with the original DEQS (mean time of 9 min 20 s vs. 9 min 19 s) [10]. The overall health status significantly correlated with the DEQS-Th’s summary scores and two subscale scores, supporting its construct validity.

This research is the first study of the psychometric properties of the DEQS questionnaire besides the original version [10], suggesting the possibility of the DEQS-Th to be applied among Thai patients. However, some limitations need to be addressed. First, despite performing the CFA, our small sample size may have affected the estimated parameters and standard error. The factor loading of each item may not have been accurate, as well as the correlated error terms. This would entail impacting the interpretation of the factor structure of the scale. A larger sample size is thus warranted. Second, other validity tests, such as criterion or predictive, should be further explored, to broaden the clinical use of the DEQS-Th. Third, this study was conducted among healthy participants with symptoms usually less severe than those presented by patients. This would have impacted the relationships between variables, resulting in different Cronbach’s alpha values and CFA results. Further studies among patients with DED are encouraged.

## Conclusion

The DEQS-Th is a promising preliminary measurement for dry eye symptoms and the impact on quality of life when tested among normal participants. Despite our small sample size, the DEQS-Th demonstrated its construct validity, reliability, and feasibility. Further studies regarding concurrent validity using other measurements, such as test-retest reliability and response to change after treatment, should be pursued.

## Supplementary Information

**Additional file 1: Table S1.** Results of the Confirmatory Factor Analysis for DEQS-Th Questionnaire

## Data Availability

The datasets used and/or analyzed during the current study available from the corresponding author upon reasonable request.

## Abbreviations

CFA: Confirmatory Factor Analysis
CFI: Comparative Fit Index
DED: Dry Eye Disease
DEQS: The Dry Eye-Related Quality-of-Life Score
DEQS-Th: The Thai Version of the Dry Eye-Related Quality-of-Life Score
IDEEL: The Impact of Dry Eye on Everyday Living
NEI VFQ-25: The 25-item National Eye Institute’s Visual Function Questionnaire
OSDI: The Ocular Surface Disease Index
QOL: Quality of Life
RMSEA: Root Mean Square Error of Approximation
TLI: Tucker–Lewis Index
WLSMV: Weighted Least Square Mean and Variance
