# Supplementary material for: Thai version of the dry eye-related quality-of-life score questionnaire: preliminary assessment for psychometric properties
Source: BMC Ophthalmol. 2021 Aug 28;21:310. doi: 10.1186/s12886-021-02077-0 (PMC8400753; doi:10.1186/s12886-021-02077-0)
Supplement: Supplementary file 1 — Additional file 1: Table S1. Results of the Confirmatory Factor Analysis for DEQS-Th Questionnaire [file 12886_2021_2077_MOESM1_ESM.docx]

Table S1. Results of the Confirmatory Factor Analysis for DEQS-Th Questionnaire

| **Item** | **Estimate** | **SE** | **Est/SE** | ***p*-Value** |
| --- | --- | --- | --- | --- |
| ***Bothersome Ocular Symptoms*** |  |  |  |  |
| 1. Foreign body sensation | 0.78 | 0.07 | 11.02 | < .001 |
| 2. Dry sensation in eyes | 0.97 | 0.06 | 15.43 | < .001 |
| 3. Painful or sore eyes | 0.51 | 0.17 | 2.95 | .003 |
| 4. Ocular fatigue | 0.83 | 0.09 | 9.78 | < .001 |
| 5. Heavy sensation in eyelids | 0.57 | 0.16 | 3.51 | < .001 |
| 6. Redness in eyes | 0.65 | 0.16 | 4.21 | < .001 |
| ***Impact on Daily Life*** |  |  |  |  |
| 7. Difficulty opening eyes | 1.08 | 0.14 | 7.73 | < .001 |
| 8. Blurred vision when watching something | 0.90 | 0.04 | 20.80 | < .001 |
| 9. Sensitivity to bright light | 0.73 | 0.08 | 9.65 | < .001 |
| 10. Problems with eyes when reading | 0.89 | 0.04 | 20.52 | < .001 |
| 11. Problems with eyes when watching television, looking at a computer or using a cell phone | 0.92 | 0.03 | 27.98 | < .001 |
| 12. Feeling distracted because of eye symptoms | 0.91 | 0.05 | 19.59 | < .001 |
| 13. Eye symptoms affecting work | 0.89 | 0.05 | 19.76 | < .001 |
| 14. Not feeling like going out because of eye symptoms | 0.73 | 0.11 | 6.42 | < .001 |
| 15. Feeling depressed because of eye symptoms | 0.83 | 0.09 | 9.05 | < .001 |

Note. SE = standard error, Est = estimated coefficient.
